# Occupational stress and psychological health impact on hypertension of miners in noisy environment in Wulumuqi, China: a case-control study

**DOI:** 10.1186/s12889-020-09760-9

**Published:** 2020-11-10

**Authors:** Yaoqin Lu, Huan Yan, Jiandong Yang, Jiwen Liu

**Affiliations:** 1 Department of Occupational and Environmental Health, College of Public Health, Xinjiang Medical University, Wulumuqi, Xinjiang China; 2 Department of Science and Education, Wulumuqi Center for Disease Control and Prevention, Wulumuqi, Xinjiang China; 3 Department of Nutrition and Food Hygiene, College of Public Health, Xinjiang Medical University, Wulumuqi, Xinjiang China; 4 Xinjiang Engineering Technology Research Center for Green Processing of Nature Product Center, Xinjiang Autonomous Academy of Instrumental Analysis, Wulumuqi, Xinjiang China; 5 Department of Tuberculosis Control and Prevention, Wulumuqi Centre for Disease Control and Prevention, Wulumuqi, Xinjiang China

**Keywords:** Occupational stress, Psychological health, Hypertension, Miners, Noisy environment

## Abstract

**Background:**

Hypertension has been declared as a global public health crisis by the World Health Organization, because of its high prevalence. It affects the health of one billion people worldwide and is directly responsible for the deaths of more than 10 million people per year. The purpose of our research was to explore the influence of occupational stress and psychological health on hypertension of miners who work in a noisy environment and provide decision reference for relevant departments to keep miners’ health.

**Methods:**

A case-control study was carried out in this research. The study subjects were divided into case groups and control groups based on whether they had hypertension or not. Effort-Reward Imbalance questionnaire and Self-Reporting Inventory questionnaire were used to investigate the psychological health status and occupational stress of the target population. General information was balanced between case and control groups through propensity score matching method. After propensity score matching, a multifactorial analysis was used to explore the impact of occupational stress and psychological health on hypertension.

**Results:**

According to the result of the multivariate analysis, psychological health was hazard to hypertension (t = 5.080, *P*<0.001) and occupational stress was not a direct risk factor for hypertension (*t* = 1.760, *P* = 0.080). The model was statistically significant (χ^2^ = 20.4, *P*<0.01).

**Conclusions:**

For miners working in the noisy environment, psychological status was a direct risk factor to hypertension, while occupational stress was an indirect factor.

## Background

Hypertension has been declared as a global public health crisis by the World Health Organization, because it has high prevalence with affecting one billion people worldwide and being directly responsible for more than 10 million deaths per year [1–3]. Therefore, many scientists have focused on hypertension studies with different directions. The research shows that the risk factors for hypertension include age, sex, lifestyles, obesity, immune and so on [4–8]. Recently, some researchers proposed that the environment has significant association with hypertension [9–11]. Meanwhile occupational stress and psychological health was proved to have influence on hypertension [12, 13]. Despite most of the research focused on the relationship between hypertension and noisy environment, noisy environment also inversely impacts occupational stress and psychological health [14, 15]. Little is known about the relationship among occupational stress, psychological health and hypertension in occupational population who work in a noisy environment. The purpose of our research was to explore the influence of occupational stress and psychological health on hypertension in miners who work in a noisy environment and provide decision reference for relevant departments to keep miners’ health.

## Methods

### Study design

The case-control study was adopted in this research. We measured sound level in workplace through the method of recommended by occupational health standards of the People’s Republic of China (GBZ/T 189.8–2007). Noisy working environment was identified based on the criteria for defining noisy working environment. People who works in a noisy workplace were included in this study, and study population was identified through inclusion criteria and exclusion criteria. The study groups were divided into case groups and control groups based on whether miners having hypertension or not in Wulumuqi. Effort-Reward Imbalance questionnaire and Self-Reporting Inventory questionnaire were used to investigate the psychological health status and occupational stress of the target population. General information was balanced between case and control groups by propensity score matching method (PSM). After propensity score matching between cases and control group, the multifactorial analysis was used to explore the impact of occupational stress and psychological health on hypertension. Structural equation method (SEM) was conducted to explore the risk factors of hypertension and for stable model selection. All participants signed the informed consent.

### Questionnaire design and content

The international general questionnaire that *Effort-Reward Imbalance* and *Self-Reporting Inventory* was adopted in this research to investigate occupational stress and psychological health respectively. Electronic questionnaire was conducted to improve the efficiency of the questionnaire. The specific contents of the survey are as follows.

### General information investigation

This section included general information, such as age, sex (Male or Female), ethnic (Han ethnic, Non-Han ethnic), educational level (Middle school, High school, Technological academy, Bachelor degree or above), working years, signed labor contract (Yes or No), professional title (No, Elementary, Middle and Senior),work shift (Day, Night and Shift work),working time per day (Less than or equal to 8 h, More than 8 h), working days per week (Less than or equal to 5 days, More than 5 days), marital status (Unmarried, Married, Dissociation, Bereft of one’s spouse) and monthly income (Less than or equal to 3000 yuan,3000-5000yuan, 5000-7000yuan, More than 7000yuan) etc.

### Occupational stress investigation

Chinese version of the *Effort-Reward Imbalance Questionnaire* adapted by Dr. Jian Li was to evaluate occupational stress. The questionnaire has 23 items belonging to three parts: External Effort (EE), Reward(R), and Overcommitment (OC). The part of EE has six items that are from item 1 to item 6, the scores range from 6 to 30, and scoring mode is positive integral. The part of R has eleven items that are from item 7 to item 17, the scores range from 11 to 55, and scoring mode is negative integral. The part of OC has six items that are from item 18 to item 23, the scores range is 6 to 30, and scoring mode is positive integral. The answer to each of the 23 items has five levels, including Not, Basically not, Sometimes, Often and Always. Extrinsic effort-reward ratio (ERI ratio) was used to identify whether imbalance exists between extrinsic effort and reward. The ERI ratio is equal to the external effect score divided by the reward score and C, and C is external effect items divided effect items. There is high input and low reward when ERI ratio greater than one, and non-high input and low reward when ERI ratio less than or equal to one [16–19].

### Psychological health investigation

Self-Reporting Inventory is one of the best-known inventories for measuring psychological health state. There are 9 dimensions (including 90 items) in Self-Reporting Inventory, and the answer to each of the 90 items has five levels, including Not, Very light, Medium, Heavier and Serious. The 9 dimensions are somatization, obsessive-compulsive symptoms, interpersonal sensitivity, depression, anxiety, hostility, phobia, paranoid ideation and psychosis, and the scores range in these dimensions are from 12 to 60, from 10 to 50, from 9 to 45, from 13 to 65, from 10 to 50, from 6 to 30, from 7 to 35, from 6 to 30 and from 10 to 50 respectively. In addition, the range of the total score for psychological health is from 90 to 450. The scoring mode are positive integral for both the 9 dimensions and the total score of psychological health. The method of calculation for each index is as follows:(1) the total score is equal to the sum of the 90 items, (2) the total average score is equal to the total score divided by 90, (3) the division of each dimension is equal to the sum of the individual dimensions divided by the number of items in each dimension, (4) number of positive items is the number of items with a total score great than or equal to 2, (5) number of negative items is the number of items with a single score of 1, (6) average score of positive symptoms is equal to the total score minus the number of negative items and divided by the number of positive items. When the total score is greater than 160, and the number of positive items is greater than 43 or the score of any factor is greater than 2, then the result is positive, and further examination is required.

### Investigation methods

Electronic questionnaire was conducted to improve the efficiency of the questionnaire. According to the Law of Prevention and Control of Occupational Diseases of the People’s Republic of China, employers should establish and improve occupational health files and workers’ health monitoring files. Therefore, we conducted questionnaires and blood pressure measurements to miners who work in a noisy workplace, when doctor conducted physical examination to them. Under the guidance of the investigators on-site, the miners who work in a noisy workplace registered into the electronic questionnaire interface through the scanned QR code by mobile phones, and filled out the questionnaire. The results can be collected by clicking on the “submit” button.

### Statistical methods and software

Continuous variable was described by mean and standard deviation if continuous variable has normal distribution, otherwise median and quartile. Categorical variable was described by constituent ratio and ratio. T-test, ANOVA, rank test, structural equation model (SEM) and propensity score method (PSM) was used to explore risk factors for hypertension of miners in a noisy condition. In this study, all analyses were based on two-sided test and the test was considered as statistically significant when *P* < 0.05. All the above processes were processed by R (Version 4.0.2).

### Quality control

Investigators who were trained and passed the examination can carry out the investigations. In order to improve the completeness of the questionnaire, each item in the electronic questionnaire was required to be answered. If questionnaire of the respondents was incomplete, the questionnaire was not used. The validity analysis of the data was completed by senior data analyst.

### Criteria

The criteria were set for obtaining more reliable results, including inclusion criteria, exclusion criteria, hypertension diagnostic criteria and criteria for defining noise working environment. The detailed content of these criteria are as follows.

### Criteria for defining a Noisy working environment

Noise was measured in the workplace according to measurement method of occupational health standards of the People’s Republic of China (GBZ/T 189.8–2007). Meanwhile, according to part 2 (physical agents) of *occupational exposure limits for hazardous agents in the workplace* in *occupational health standards of the People’s Republic of China (GBZ 2.2–2007)*, noise was defined in workplace environment. The detailed content of the criteria are as follows:

Miners exposed to an equivalent noise level of more than 85 dB(A) for 8 h per day and 5 days per week were eligible for the study.

### Hypertension diagnostic criteria

According to the Guidelines for Prevention and Treatment of Hypertension in China (2018), hypertension was diagnosed when the systolic blood pressure was greater than or equal to 140 mmHg and the diastolic blood pressure was greater than or equal to 90 mmHg. Moreover, participants were asked to sit for at least 10 min before measurement. The right upper arm blood pressure was measured twice by Omron calibrated electronic sphygmomanometer for each subject, and result was reported as the average of the two measurements.

### Inclusion criteria and exclusion criteria

All workers who work in the noisy workplace were incorporated into the study. The exclusion criteria were (1) those who had serious illness (except hypertension), (2) those who did not agree to participate in the investigation, (3) the questionnaire was invalid.

## Results

### Sample collection

**Fig. 1:**
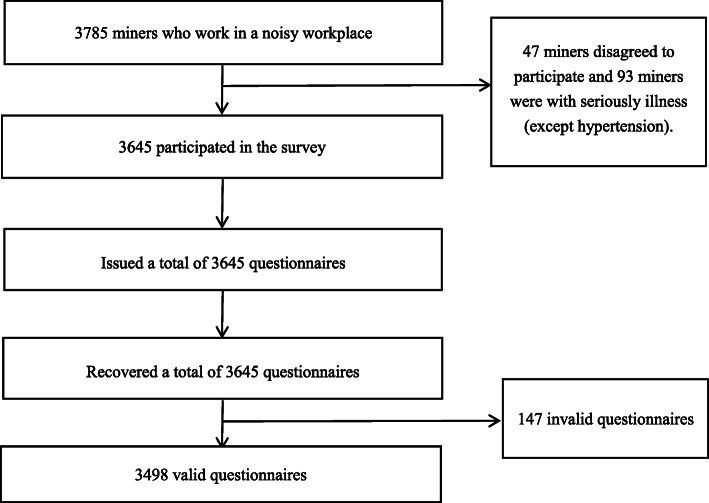
Flowchart of sample collection

There were 3785 miners who work in a noisy workplace in Wulumuqi. However, 47 miners among the 3785 miners disagreed to participate the survey and 93 miners had serious illness (except hypertension). Therefore, after excluding the miners who disagreed to participate and who were seriously sick, we issued a total of 3645 questionnaires to miners e, and the participation rate was 96.30% (3645/3785). Finally, we collected 3645 questionnaires, and of which 3498 were valid questionnaires. Response rate and complete response rate was 100% (3645/3645) and 95.97% (3498/3645) respectively (Fig. 1).

### Demographic characteristics of the population

Tables 1 and 2 shows the demographic characteristics of the case (Hypertension = Yes) and the control group (Hypertension = No). The median of age was 44 and 48 years old, and the median of working years was 24 and 29 years in the case and the control group respectively. Among the 3498 miners, 2484 were men (71.01%) and 1014 were women (28.99%). The number of miners with occupational stress was high in both the case and control group, but there was more psychological health problem in the case group than the control group. In addition, the distribution of professional title, work shift, marital status and monthly income was generally consistent in both groups.

**Table 1:** Description of continuous variables

	Hypertension = No		Hypertension = Yes	
	*N*	*M*(*Q*_*25*_,*Q*_*75*_)	*N*	*M*(*Q*_*25*_,*Q*_*75*_)
Age	2618	44 (34,49)	880	48 (45,51)
Working years	2618	24 (10,29)	880	29 (24,32)

**Table 2:** General information of categorical data

	Hypertension = No		Hypertension = Yes		Total	
	Numbers (*N*)	Constituent Ratio (*%*)	Numbers (*N*)	Constituent Ratio (*%*)	Numbers *(N*)	Constituent Ratio (*%*)
Sex						
Male	1678	64.09	806	91.59	2484	71.01
Female	940	35.91	74	8.41	1014	28.99
Ethnic						
Han ethnic	2179	83.23	768	87.27	2947	84.25
Non-Han ethnic	439	16.77	112	12.73	551	15.75
Educational level						
Middle school	109	4.16	38	4.32	147	4.20
High school	496	18.95	287	32.61	783	22.38
Technological academy	1215	46.41	389	44.20	1604	45.85
Bachelor degree or above	798	30.48	166	18.86	964	27.56
Sign labor contract						
Yes	2596	99.16	879	99.89	3475	99.34
No	22	0.84	1	0.11	23	0.66
Professional title						
No	974	37.20	341	38.75	1315	37.59
Elementary	524	20.02	97	11.02	621	17.75
Middle	634	24.22	230	26.14	864	24.70
Senior	486	18.56	212	24.09	698	19.95
Work shift						
Day	1310	50.04	457	51.93	1767	50.51
Night	83	3.17	26	2.95	109	3.12
Shift work	1225	46.79	397	45.11	1622	46.37
Working time per day						
≤ 8	1830	69.90	631	71.70	2461	70.35
>8	788	30.10	249	28.30	1037	29.65
Working days per week						
≤ 5	1970	75.25	641	72.84	2611	74.64
>5	648	24.75	239	27.16	887	25.36
Marital status						
Unmarried	282	10.77	42	4.77	324	9.26
Married	2160	82.51	765	86.93	2925	83.62
Dissociation	162	6.19	65	7.39	227	6.49
Bereft of one’s spouse	14	0.53	8	0.91	22	0.63
Monthly income						
≤ 3000 Yuan	708	27.04	254	28.86	962	27.50
3000 ~ 5000 Yuan	1476	56.38	509	57.84	1985	56.75
5000 ~ 7000 Yuan	377	14.40	102	11.59	479	13.69
>7000 Yuan	57	2.18	15	1.70	72	2.06
Occupational Stress						
No	1279	48.85	359	40.80	1638	46.83
Yes	1339	51.15	521	59.20	1860	53.17
Psychological health problem						
No	1527	58.33	355	40.34	1882	53.80
Yes	1091	41.67	525	59.66	1616	46.20

### Confounding factor analysis

To control the confounding factors and eliminate the bias of general demographic characteristics, the propensity score method (PSM) was used to select a total of 12 factors as the matching covariates, including age, sex, ethnic, educational level, working years, signed labor contract or not, professional title, work shift, working time per day, working days per week, marital status and monthly income. The caliper value and random seed were set at 0.02 and 1 respectively and 1:1 proximity matching method was used. Hypertension and general demographic characteristics were defined as the dependent variable and independent variables respectively. Multiple logistic regression analysis was conducted to explore the efficiency of PSM. Before matching, there were statistically significant differences in age, sex, working years (*P* < 0.001). After matching, there were no statistically significant differences among characteristics of participants between the case group and the control group (*P* > 0.05) (Table 3).

**Table 3:** Analysis of propensity score

Factor	*β* (*CI95%*)	*OR* (*CI95%*)	*t*	*P*	*VIF*
Before					
Intercept	0.61 (−1.69,2.90)	1.84 (0.18,18.26)	0.520	0.610	–
Age	0.02 (0.01,0.04)	1.02 (1.01,1.04)	2.870	**<0.001**	2.450
Sex	−1.64 (− 1.90,-1.38)	0.19 (0.15,0.25)	−12.360	**<0.001**	1.050
Ethnic	−0.15 (− 0.39,0.09)	0.86 (0.68,1.10)	−1.190	0.230	1.030
Educational level	− 0.03 (− 0.14,0.09)	0.97 (0.87,1.09)	− 0.480	0.630	1.270
Working years	0.03 (0.02,0.05)	1.03 (1.02,1.05)	5.150	**<0.001**	2.550
Sign labor contract	−1.62 (−3.70,0.45)	0.20 (0.02,1.58)	− 1.530	0.130	1.000
Professional title	−0.02 (− 0.09,0.05)	0.98 (0.92,1.05)	− 0.560	0.580	1.040
Work shift	0.07 (−0.03,0.16)	1.07 (0.97,1.17)	1.410	0.160	1.230
Working time per day	0.05 (−0.15,0.24)	1.05 (0.86,1.27)	0.460	0.640	1.160
Working days per week	0.05 (−0.14,0.24)	1.05 (0.87,1.27)	0.510	0.610	1.030
Marital status	0.18 (−0.04,0.40)	1.20(0.96,1.49)	1.630	0.100	1.090
Monthly income	−0.09 (− 0.22,0.03)	0.91 (0.80,1.03)	−1.460	0.140	1.160
After					
Intercept	−13.26 (− 649.75,623.23)	0.00 (0.00,4.6e+ 270)	− 0.040	0.970	–
Age	0.02 (0.00,0.04)	1.02 (1.00,1.05)	2.190	0.030	2.770
Sex	0.12 (−0.24,0.47)	1.13 (0.79,1.60)	0.650	0.520	1.050
Ethnic	−0.01 (− 0.29,0.28)	0.99 (0.75,1.32)	−0.050	0.960	1.020
Educational level	0.01 (−0.12,0.14)	1.01 (0.89,1.15)	0.130	0.900	1.260
Working years	−0.01 (− 0.03,0.01)	0.99 (0.97,1.01)	−1.100	0.270	2.780
Sign labor contract	12.6 (−623.89,649.08)	296,558.57 (0.00,7.83e+ 281)	0.040	0.970	1.000
Professional title	0 .00(−0.08,0.08)	1.00 (0.93,1.08)	0.080	0.940	1.030
Work shift	0.04 (−0.06,0.15)	1.04 (0.94,1.16)	0.800	0.420	1.220
Working time per day	0.02 (−0.21,0.24)	1.02 (0.81,1.27)	0.140	0.890	1.150
Working days per week	0.01 (−0.21,0.23)	1.01 (0.81,1.25)	0.090	0.930	1.030
Marital status	−0.23 (− 0.47,0.02)	0.79 (0.62,1.02)	−1.810	0.070	1.050
Monthly income	0.02 (−0.13,0.16)	1.02 (0.88,1.18)	0.220	0.820	1.110

### Analysis of influencing factors

After balancing the general information between case and control group, we used a multi factorial analysis to explore the impact of occupational stress and psychological health on hypertension. The model was statistically significant (*χ2* = 20.4, *P*<0.01). According to the result of the multivariate analysis, psychological health is hazards to hypertension (*t* = 5.080,*P*<0.001). However, the occupational stress was not significantly associated with hypertension (*t* = 1.760, *P* = 0.080). (Table 4).

**Table 4:** Effects of psychological and occupational stress on hypertension

Factor	*β* (*CI95%*)	*OR* (*CI95%*)	*t*	*P*	*VIF*
Intercept	− 0.37 (− 0.53,-0.21)	0.69 (0.59,0.81)	−4.470	<0.001	–
Psychological health	0.51 (0.31,0.71)	1.67 (1.37,2.03)	5.080	<0.001	1.070
Occupational Stress	0.18 (−0.02,0.38)	1.2 (0.98,1.46)	1.760	0.080	1.070

Structural equation method was then conducted to explore the influencing factors for hypertension and for stable model selection. The criteria for a stable model are shown in Table 5. The stable SEM model was selected by variables selection, model fitting and model validation. The model adjustment parameter is as in Table 6.

**Table 5:** Criteria for a stable SEM model

Index	Recommend value	Acceptance
χ^2^/df	< 3 good fit < 5 reasonable fit	Good
RMSEA	< 0.05 good fit < 0.10 reasonable fit	Reasonable
NFI	Above 0.9	Good
NNFI	Above 0.9	Good
CFI	Above 0.9	Good
IFI	Above 0.9	Good
SRMR	< 0.05 good fit < 0.10 reasonable fit	Reasonable

**Table 6:** Comparison of models before and after correction

	Before adjustment	After adjustment
*RMR*	14.197	0.204
*GFI*	0.792	0.990
*AGFI*	0.464	0.969
*IFI*	0.194	0.976
*CFI*	0.192	0.976
*RMSEA*	0.306	0.057
*PCLOSE*	0.000	0.067

**Fig. 2:**
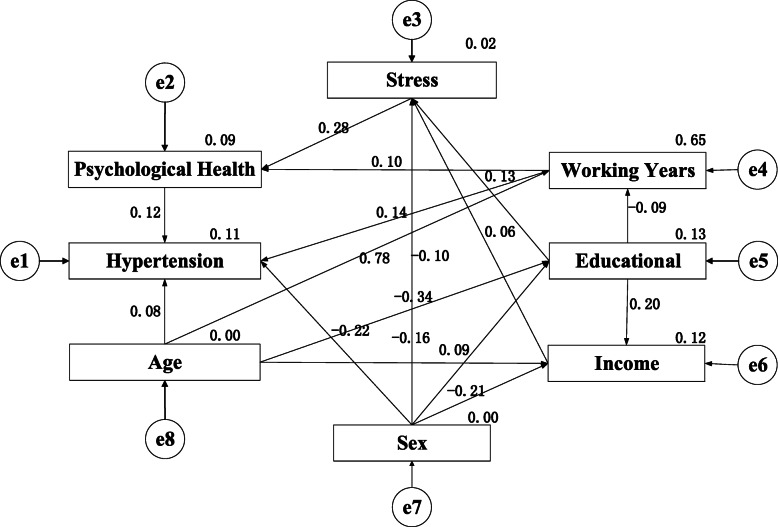
Path diagram

The final model path diagram is shown in Fig. 2. It can be seen that the influence of stress on hypertension was through psychological health. In other words, stress has no direct influence on hypertension. In addition, according to the path diagram, psychological health, working years, sex and age show direct influence on hypertension. At the same time, sex and age also show indirect influence on stress. Moreover, educational and income have only one unidirectional pathway to influence hypertension, which indirectly affects hypertension through stress.

With regard to the statistical test, each path in the path diagram shows statistical significance (*P*<0.05) (Table 7).

**Table 7:** Model parameter test

Path	*Standardized Estimate*	*S.E.*	*C.R*.	*P*
Educational level←Sex	0.093	0.029	5.784	<0.001
Educational level←Age	−0.341	0.001	−21.318	<0.001
Monthly income←Age	−0.156	0.001	−9.192	<0.001
Monthly income←Educational level	0.203	0.015	11.994	<0.001
Monthly income←Sex	−0.211	0.025	−13.131	<0.001
Occupational Stress←Sex	−0.101	0.019	−5.830	<0.001
Working Years←Age	0.776	0.012	73.027	<0.001
Working Years←Educational level	−0.086	0.143	−8.147	<0.001
Occupational Stress←Monthly income	−0.057	0.013	−3.226	0.001
Occupational Stress←Educational level	0.134	0.011	7.673	<0.001
Psychological health←Occupational Stress	0.283	0.016	17.542	<0.001
Psychological health←Working years	0.102	0.001	6.334	<0.001
Hypertension←Sex	−0.219	0.015	−13.496	<0.001
Hypertension←Working years	0.141	0.001	5.213	<0.001
Hypertension←Psychological health	0.124	0.014	7.763	<0.001
Hypertension←Age	0.081	0.001	3.025	0.002

## Discussion

In this study, we investigated 3785 miners whose work exposed to a noisy environment in Wulumuqi. Cluster sampling is used in the study. The results discovered the relationship among occupational stress, psychological health and hypertension in miners whose work exposed to a noisy environment in Wulumuqi.

The study found that the demographic characteristics of the population, except sex, occupational stress and psychological health, was not significantly different between the case and control group. Similar to other hard-labor working environment, it is a common phenomenon that there are more male than female miners [20]. Level of occupational stress and psychological health was different between the case and control group, indicating that occupational stress, psychological health and hypertension were correlated [21, 22].

Propensity score matching (PSM) is a statistical matching technique that attempts to estimate the effect of a treatment and intervention by accounting for the covariates that predict the treatment effects. It attempts to reduce the bias due to confounding variables that could be found in estimating the treatment effect from simply comparing outcomes among groups that received the treatment versus those that did not [23]. This method can adjust covariates more objectively and scientifically and thereby get more intuitive conclusions.

We analyzed the influence of occupational stress and psychological health on hypertension after controlling other factors through propensity score matching. The result showed that psychological health was one of the risk factors for hypertension, which was consistent with the study reported by Tevie J’s [24]. Psychological health induced hypertension may relate to the sympathetic nervous system. Jeanie Park’s study showed that psychological activities can directly affect the brain to influence the body’s catecholamine secretion, thereby affecting the sympathetic nerve excitability [25]. Sympathetic excitation is one of the main physiological causes of hypertension [26–28]. The results showed no direct relationship between occupational stress and hypertension, after balancing other factors through propensity score matching. To our knowledge, this is the first study to show such a view compared to previous studies [29, 30].

Structural equation method was used to further explore the influence factors of hypertension. The results showed that occupational stress had an indirect effect on hypertension. Stress that can cause the body to produce a stress response was proved to be the main reason for hypertension [31–33]. Occupational stress is one type of stress. Therefore, occupational stress can cause the body to produce a stress response too. Some researchers believe that under stress the level of certain hormones in our body will increase. Some of these hormones will affect our psychological condition, including norepinephrine, adrenaline, adrenal cortex hormones, etc. [34, 35]. Therefore, the impact of occupational stress on hypertension may be caused by changes in psychological state, which was caused by increased secretion of certain hormones in the body under stress. Research shows that noise is one of the causes of occupational stress [36, 37]. Therefore, working in a noisy environment for a long time will lead to increased occupational pressure on workers, and these workers will be more prone to hypertension.

Through the analysis of the path map, we found that psychological condition, working years, sex and age of miners working in the noisy environment have direct influence on hypertension. The results are consistent with some previous studies [38, 39]. Interestingly, we found that age and gender have both direct or indirect effects on occupational stress or psychological health. This may be related to the stress and psychological tolerance of different age and gender groups. In addition, income and education was also found to affect occupational stress. Some researchers believe that the more educated, the less stressed [39]. At the same time, studies show that low income was also a risk factor for occupational stress [40–43]. Not in the same way as education and income, working years directly affects psychological status and leads to high blood pressure. Facing the problems of career development, family life, children’s education, employment and marriage, as well as various events affecting work and life, psychological problems may occur accompanied [44–46].

In summary, case control design was adopted in this study to explore the influence of occupational stress and psychological health on hypertension in miners whose work is exposed in a noisy environment. Through propensity score matching method to minimize confounding bias and achieve comparable improvement, the factors influencing hypertension of miners in a noisy environment were scientifically investigated. However, retrospective studies have limitations in data quality because the results were relied on memory, therefore studies are treated in a low position in the hierarchy of evidence. To further verify the causality, prospective research needs to be conducted.

## Conclusion

In a noisy working environment, the psychological condition of miners will directly affect the occurrence of hypertension. However, the impact of occupational stress on hypertension is achieved through affecting the psychological state.

## Data Availability

The datasets used and/or analyzed during the current study are available from the corresponding author on reasonable request.

## Abbreviations

VIF: Variance Inflation Factor
ERI: Effort-Reward Imbalance
EE: External Effort
OC: Overcommitment
SEM: Structural Equation Model
ANOVA: Analysis of Variance
PSM: Propensity Score Method
